# Supplementary material for: Proteome and phosphoproteome signatures of recurrence for HPV+ head and neck squamous cell carcinoma
Source: Commun Med (Lond). 2022 Jul 30;2:95. doi: 10.1038/s43856-022-00159-8 (PMC9338924; doi:10.1038/s43856-022-00159-8)
Supplement: Supplementary file 1 — Supplementary Information [file 43856_2022_159_MOESM1_ESM.pdf]

Supplementary Information

Proteome and phosphoproteome signatures of recurrence for HPV^{+} head and neck squamous cell carcinoma

Tomonori Kaneko, Peter YF. Zeng, Xuguang Liu, Rober Abdo, John W. Barrett, Qi Zhang, Anthony C. Nichols and Shawn Shun-Cheng Li

Supplementary Table 1. Clinical information of patients

Sample ID	gender	age at diagnosis	smoking	Sample classification	Primary or secondary tumor	Patient recurred?	hpv	DFS	dfs 60mon (treatment to recurrence for recurrence samples)	Death 60mon	os 60mon	subsite	subsite 2	site 1 oral cavity 2 oropharynx 3 larynx 4 hp 5 other	t stage	n stage	last known status	date of last known status	date of recurrence	date of 2nd recurrence	site of recurrence	Treatment (C: cisplatin; S: Surgery; RT: Radiation)
NR1	male	50-59	Yes	no recurrence	Primary	No	HPV16	0	60	0	60	Tonsil	tonsil	2	2 or 3	2 or 3	AWOD	5/19/2017	NA	NA	NA	CRT
NR2	male	60-69	No	no recurrence	Primary	No	HPV16	0	60	0	60	BOT	BOT	2	2 or 3	2 or 3	AWOD	3/9/2018	NA	NA	NA	CRT
NR3	male	40-49	No	no recurrence	Primary	No	HPV16	0	57	0	57	Tonsil	tonsil	2	2 or 3	2 or 3	AWOD	1/13/2017	NA	NA	NA	CRT
NR4	male	40-49	No	no recurrence	Primary	No	HPV16	0	60	0	60	Tonsil	tonsil	2	2 or 3	2 or 3	AWOD	7/5/2018	NA	NA	NA	CRT
NR5	male	50-59	No	no recurrence	Primary	No	HPV16	0	52	0	52	Tonsil	tonsil	2	0 or 1	0 or 1	AWOD	1/9/2017	NA	NA	NA	S + RT
NR6	male	60-69	Yes	no recurrence	Primary	No	HPV16	0	11	0	11	Tonsil	tonsil	2	2 or 3	0 or 1	AWOD	9/25/2014	NA	NA	NA	S + RT
NR7	male	40-49	Yes	no recurrence	Primary	No	HPV16	0	56	0	56	Tonsil	tonsil	2	0 or 1	2 or 3	AWOD	7/17/2018	NA	NA	NA	S + RT
NR8	male	70-79	Yes	no recurrence	Primary	No	HPV16	0	47	0	47	Tonsil	tonsil	2	0 or 1	2 or 3	AWOD	9/27/2019	NA	NA	NA	CRT
R1	male	60-69	Yes	future recurrence	Primary	Yes	HPV16	1	24	1	31	SP	SP	2	2 or 3	0 or 1	DWD	4/14/2014	9/6/2013	NA	local (pharynx)	CRT
R2	male	70-79	Yes	future recurrence	Primary	Yes	HPV16	1	11	1	24	BOT	BOT	2	2 or 3	2 or 3	DOD	9/13/2018	8/15/2017	NA	local, lungs	CRT
R3	male	60-69	No	future recurrence	Primary	Yes	HPV16	1	5	0	13	Tonsil	tonsil	2	0 or 1	2 or 3	AWOD	10/4/2019	2/4/2019	8/26/2019	local and regional	CRT
R4	female	50-59	Yes	relapsed	Secondary	Yes	HPV16	1	21	1	26	BOT	BOT	2	2 or 3	0 or 1	DWD	6/22/2013	1/22/2013	NA	locoregional (BOT and bil neck)	CRT
R5	male	80-89	Yes	relapsed	Secondary	Yes	HPV16	1	5	1	6	Tonsil	tonsil	2	2 or 3	0 or 1	DWOD	4/22/2013	3/5/2013	NA	local	RT
R6	male	60-69	No	relapsed	Secondary	Yes	HPV16	1	13	1	31	BOT	BOT	2	2 or 3	0 or 1	DOD	5/5/2018	11/25/2016	3/16/2017	local	CRT
R7	male	50-59	Yes	relapsed	Secondary	Yes (>60 months)	HPV16	0	60	0	60	Tonsil	NA	2	0 or 1	2 or 3	AWOD	8/14/2019	2/19/2019	NA	right neck 2010, then left base of tongue 2019	CRT

Supplementary Table 2

Arrangement of the samples in the three TMT-11plex batches

**Channel**	**TMT-A**	**TMT-B**	**TMT-C**
**1**	Boost	Boost	Boost
**2**	R6	NR4	C5
**3**	**Pool**	**Pool**	**Pool**
**4**	R3	NR1	**C2**
**5**	C1	R7	R6
**6**	R4	**C2**	C4
**7**	**C2**	R5	R3
**8**	NR5	C3	R5
**9**	R2	NR8	NR4
**10**	NR2	C5	R1
**11**	NR6	NR3	NR7

Note: The technical triplicate of sample C2 is placed across the three batches, which was used for TMT batch correction of the pTyr datasets. The pool channel was used for TMT batch correction of the proteome and IMAC datasets.

Supplementary Table 3

Mass spectrometry data collection parameters

	Proteome	IMAC phospho-proteome	Superbinder phospho-proteome
LC gradient (min)	4 hours	4 hours	1 hour
MS1 resolution	70000	140000	70000
MS1 AGC	3E+06	3E+06	3E+06
MS1 maxIT (ms)	50	50	50
MS1 mass range (m/z)	375 to 1400	375 to 1400	375 to 1400
MS2 resolution	70000	140000	140000
MS2 AGC	1E+05	1E+05	1E+05
MS2 maxIT (ms)	240	500	500
TopN	10	6	10
Isolation window (m/z)	0.7	0.7	0.7
Fixed first mass (m/z)	120	120	120
NCE	32	32	32
Dynamic exclusion (s)	50	50	30

Supplementary Table 4

A list of top 40 significantly overexpressed proteins in the recurrent tumors

Manhattan distance	-log (p-value)	log2 difference	Gene	Protein names
7.4	4.7	2.7	FGG	Fibrinogen gamma chain
7.4	5.1	2.3	MMP8	Neutrophil collagenase (EC 3.4.24.34) (
7.2	4.6	2.6	LTF	Kaliocin-1;Lactoferroxin-A;Lactoferrox
7.2	4.7	2.5	FGB	Fibrinogen beta chain [Cleaved into: Fi
7.2	4.4	2.8	CAMP	Antibacterial peptide LL-37];Cathelicid
7.2	4.8	2.4	MMP9	82 kDa matrix metalloproteinase-9];M
7.1	4.6	2.5	C4BPA	C4b-binding protein alpha chain (C4bp
7.1	5.2	1.8	LYZ	Lysozyme C (EC 3.2.1.17) (1,4-beta-N-a
7.0	4.6	2.3	PRTN3	Myeloblastin (EC 3.4.21.76) (AGP7) (C-
6.8	4.0	2.8	BPI	Bactericidal permeability-increasing pr
6.8	4.7	2.1	F2	Activation peptide fragment 2;Prothro
6.8	4.6	2.2	C4BPB	C4b-binding protein beta chain
6.5	4.2	2.3	C9	Complement component C9 [Cleaved i
6.5	4.2	2.3	ELANE	Neutrophil elastase (EC 3.4.21.37) (Bor
6.5	3.6	2.9	DEFA3	Neutrophil defensin 2 (HNP-2) (HP-2) (H
6.4	4.2	2.2	C5	C5a anaphylatoxin;Complement C5 (C3
6.4	4.7	1.7	PROS1	Vitamin K-dependent protein S
6.4	4.1	2.3	LBP	Lipopolysaccharide-binding protein (LI
6.2	4.0	2.2	MPO	84 kDa myeloperoxidase;89 kDa myelo
6.2	4.0	2.2	C8B	Complement component C8 beta chair
6.2	4.3	1.9	PTX3	Pentraxin-related protein PTX3 (Pentax
6.2	4.3	1.8	ORM1	Alpha-1-acid glycoprotein 1 (AGP 1) (Or
6.2	4.1	2.1	C6	Complement component C6
6.2	4.1	2.1	ARG1	Arginase-1 (EC 3.5.3.1) (Liver-type argi
6.1	4.4	1.8	CDA	Cytidine deaminase (EC 3.5.4.5) (Cytidi
6.1	3.8	2.4	CRP	C-reactive protein [Cleaved into: C-reac
6.1	4.2	1.9	CP	Ceruloplasmin (EC 1.16.3.1) (Ferroxida
6.0	3.8	2.2	CFHR2	Complement factor H-related protein 2
6.0	3.9	2.1	FGA	Fibrinogen alpha chain [Cleaved into: F
5.9	3.7	2.3	C7	Complement component C7
5.9	3.4	2.5	CRISP3	Cysteine-rich secretory protein 3 (CRISI
5.9	3.8	2.1	ANXA3	Annexin A3 (35-alpha calcimedin) (Ann
5.9	3.5	2.4	COL12A1	Collagen alpha-1(XII) chain
5.8	3.9	2.0	C8A	Complement component C8 alpha cha
5.8	4.1	1.8	ITIH4	35 kDa inter-alpha-trypsin inhibitor he
5.7	4.2	1.6	ITIH3	Inter-alpha-trypsin inhibitor heavy cha
5.7	3.4	2.3	S100P	Protein S100-P (Migration-inducing ge
5.7	3.6	2.1	C1QA	Complement C1q subcomponent subu
5.7	3.8	2.0	PLG	Activation peptide;Angiostatin;Plasmi
5.7	3.4	2.2	AZU1	Azurocidin (Cationic antimicrobial pro

Note: A list of the proteins that are significantly more abundant in the recurrent (R), compared to the non-recurrent (NR), tumors, sorted by the Manhattan distance ($-\log_{10}(p) + \log_2$ difference). The top 40 proteins are shown. n = 8 for the NR group, and n = 7 for the R group.

Supplementary Table 5

A list of 23 significantly under-expressed proteins in the recurrent tumors

Manhattan distance	-log (p-value)	log2 difference	Gene	Protein names
6.6	4.8	-1.8	CD2	T-cell surface antigen CD2 (Erythrocyte
5.2	3.4	-1.8	CD6	T-cell differentiation antigen CD6 (T12)
5.2	3.9	-1.2	CD8A	T-cell surface glycoprotein CD8 alpha c
4.8	3.5	-1.2	CD3G	T-cell surface glycoprotein CD3 gamma
4.7	3.5	-1.2	H2AC4	Histone H2A type 1-B/E (Histone H2A.2
4.6	3.6	-1.0	SUPT4H1	Transcription elongation factor SPT4 (h
3.8	2.4	-1.4	SP4	Transcription factor Sp4 (SPR-1)
3.7	1.6	-2.0	FAM3B	Protein FAM3B (Cytokine-like protein 2
3.6	2.3	-1.3	CD5	T-cell surface glycoprotein CD5 (Lymph
3.6	2.4	-1.2	FOXE1	Forkhead box protein E1 (Forkhead box
3.6	2.5	-1.1	LGALS9	Galectin-9 (Gal-9) (Ecalectin) (Tumor an
3.4	2.1	-1.3	RBP5	Retinol-binding protein 5 (Cellular reti
3.1	1.4	-1.7	RALGAPA2	Ral GTPase-activating protein subunit a
3.0	1.6	-1.5	LMO4	LIM domain transcription factor LMO4
2.8	1.6	-1.2	PTN	Pleiotrophin (PTN) (Heparin-binding br
2.7	1.3	-1.3	SMO	Smoothened homolog (SMO) (Protein G
2.6	1.4	-1.2	CCR6	C-C chemokine receptor type 6 (C-C CKR
2.5	1.2	-1.3	CRIPT	Cysteine-rich PDZ-binding protein (Cys
2.4	1.3	-1.1	SSBP3	Single-stranded DNA-binding protein 3
2.3	1.2	-1.1	TMEM59	Transmembrane protein 59 (Liver mem
2.3	1.2	-1.1	TMPRSS11B	Transmembrane protease serine 11B (E
2.2	1.2	-1.0	RHCG	Ammonium transporter Rh type C (Rh g
2.2	1.0	-1.2	KRT13	Keratin, type I cytoskeletal 13 (Cytoker

Note: Shown above is a list of 23 proteins that are significantly less abundant in the recurrent (R) tumors, compared to the non-recurrent (NR) tumors, according to the Volcano plot in Fig. 1. n = 8 for the NR group, and n = 7 for the R group.

Supplementary Table 6

A list of pTyr sites with significant changes between recurrent and non-recurrent tumors

log2 (recurrence / no recurrence)	boost / sample ratio	pTyr site	Protein names
2.6	1.3	COL11A1_Y360	Collagen alpha-1(XI) chain
1.7	6.9	FGG_Y448	Fibrinogen gamma chain
1.6	2.4	VTN_Y75	Somatomedin-B];Vitronectin (VN) (S-
1.6	190.3	KATNB1_Y382	Katanin p80 WD40 repeat-containing
1.5	18.1	SIT1_Y188	Signaling threshold-regulating trans
1.4	4.9	STAT5A_Y694	Signal transducer and activator of tr
1.4	134.1	ITGB4_Y1492	Integrin beta-4 (GP150) (CD antigen
1.4	27.3	CASS4_Y174	Cas scaffolding protein family meml
1.4	1.6	SERPIND1_Y79	Heparin cofactor 2 (Heparin cofacto
1.3	1.2	GNAI3_Y195	Guanine nucleotide-binding protein (
1.2	1.5	ANXA1_Y39	Annexin A1 (Annexin I) (Annexin-1) ((
1.2	35.1	OTUD4_Y460	OTU domain-containing protein 4 (E
1.1	4.5	MAPK9_Y185	Mitogen-activated protein kinase 9 (
1.0	92.2	RAB34_Y247	Ras-related protein Rab-34 (Ras-rela
-1.0	237.1	SLC12A6_Y122	Solute carrier family 12 member 6 (
-1.0	41.3	DENND2B_Y488	DENN domain-containing protein 2B
-1.0	8.1	SPTAN1_Y2430	Spectrin alpha chain, non-erythrocyt
-1.0	166.6	MAPRE1_Y124	Microtubule-associated protein RP/E
-1.0	26.3	ARHGEF16_Y216	Rho guanine nucleotide exchange fa
-1.0	1.2	CCNL1_Y376	Cyclin-L1 (Cyclin-L)
-1.0	17.0	EXPH5_Y1019	Exophilin-5 (Synaptotagmin-like prot
-1.1	135.9	ANXA1_Y21	Annexin A1 (Annexin I) (Annexin-1) ((
-1.1	3.8	SRP14_Y27	Signal recognition particle 14 kDa p
-1.1	97.6	PLEKHA7_Y665	Pleckstrin homology domain-contain
-1.1	5.9	CR2_Y1029	Complement receptor type 2 (Cr2) ((
-1.1	51.0	CMIP_Y752	C-Maf-inducing protein (c-Mip) (Tru
-1.1	20.7	MYO1E_Y971	Unconventional myosin-Ie (Myosin-I
-1.1	26.1	PKP1_Y187	Plakophilin-1 (Band 6 protein) (B6P)
-1.1	6.0	PKM_Y175	Pyruvate kinase PKM (EC 2.7.1.40) ((
-1.1	58.5	CTNND1_Y174	Catenin delta-1 (Cadherin-associated
-1.1	15.0	TMEM192_Y213	Transmembrane protein 192
-1.1	37.4	CTNND1_Y96	Catenin delta-1 (Cadherin-associated
-1.1	97.2	CD247_Y83	T-cell surface glycoprotein CD3 zeta
-1.2	209.3	PKP2_Y119	Plakophilin-2
-1.2	2.3	LCK_Y192	Tyrosine-protein kinase Lck (EC 2.7.1
-1.2	41.2	SYK_Y323	Tyrosine-protein kinase SYK (EC 2.7.
-1.2	92.0	MATR3_Y250	Matrin-3
-1.2	11.5	ENO1_Y44	Alpha-enolase (EC 4.2.1.11) (2-phosp
-1.2	75.4	C6orf132_Y1017	Uncharacterized protein C6orf132
-1.2	72.5	PKP1_Y214	Plakophilin-1 (Band 6 protein) (B6P)
-1.3	1.4	KIAA1217_Y244	Sickle tail protein homolog
-1.3	72.9	EPHA2_Y588	Ephrin type-A receptor 2 (EC 2.7.10.1
-1.3	24.3	PKP1_Y160	Plakophilin-1 (Band 6 protein) (B6P)
-1.3	1090.9	TBCB_Y98	Tubulin-folding cofactor B (Cytoskele
-1.3	1.3	LCP1_Y28	Plastin-2 (L-plastin) (LC64P) (Lympho
-1.3	0.9	SPTBN1_Y1680	Spectrin beta chain, non-erythrocytic
-1.4	11.3	ACTB_Y53	Actin, cytoplasmic 1 (Beta-actin) [Cle
-1.4	5.7	LYN_Y473	Tyrosine-protein kinase Lyn (EC 2.7.1
-1.5	2.7	VAV1_Y826	Proto-oncogene vav
-1.5	4.0	ITSN2_Y553	Intersectin-2 (SH3 domain-containin
-1.5	54.6	NECTIN2_Y513	Nectin-2 (Herpes virus entry mediato
-1.7	65.3	CD3G_Y160	T-cell surface glycoprotein CD3 gamn
-1.7	2.3	SPRR3_Y138	Small proline-rich protein 3 (22 kDa
-2.0	11.4	LCK_Y505	Tyrosine-protein kinase Lck (EC 2.7.1
-2.3	0.7	GART_Y348	Phosphoribosylformylglycinamidine
-4.0	2.5	MYH2_Y1381	Myosin-2 (Myosin heavy chain 2) (M

Note: the boost/sample ratio is a median intensity ratio between the pervanadate-treated boost channels and sample channels. The pTyr sites with a boost/sample ratio greater than 100 are shaded in grey.

Supplementary Table 7

The phosphosite data used to draw the ECM-receptor interaction KEGG pathway

Phosphosite	log2 (recurrence / no recurrence)	N: -Log10 (p-value)	PhosphositePlus_FUNCTION	PhosphositePlus_PROCESS
**ITGA4_S1021**	-0.94	1.29	molecular association, regulation; phosphorylation;activity, induced	cell growth, inhibited; cell motility, altered; cell motility, induced; cytoskeletal reorganization;carcinogenesis, induced
**SDC4_Y197**	-0.68	2.05		
**CD44_S697**	-0.18	0.22		cell motility, altered
**ITGA3_Y1028**	-0.10	0.13		
**ITGB1_Y783**	-0.05	0.10	intracellular localization;molecular association, regulation	
**ITGA6_Y1110**	0.05	0.05		
**DAG1_T790**	0.25	0.34		
**ITGB3_Y773**	0.31	0.81	activity, inhibited; intracellular localization; molecular association, regulation; protein conformation; protein processing;activity, induced	cell adhesion, altered; cell motility, altered; cytoskeletal reorganization;cell adhesion, induced
**FN1_S2475**	0.67	0.64		
**SDC1_Y286**	0.77	1.76		endocytosis, induced
**ITGA5_S127**	1.09	0.85		
**GP1BB_S191**	1.12	0.72	receptor desensitization, altered;molecular association, regulation	cell adhesion, altered
**GP1BA_S651**	1.34	1.37	molecular association, regulation	
**TNC_S72**	1.39	1.43		
**ITGB4_Y1492**	1.43	1.89	molecular association, regulation	
**VTN_Y75**	1.59	2.91		
**COL6A3_S1783**	1.99	1.86		

Note: If multiple phosphosites were identified for a given protein, the phosphosite with known function or showing higher statistical significance between the non-recurrence (NR) and recurrence (R) groups was selected for the protein.

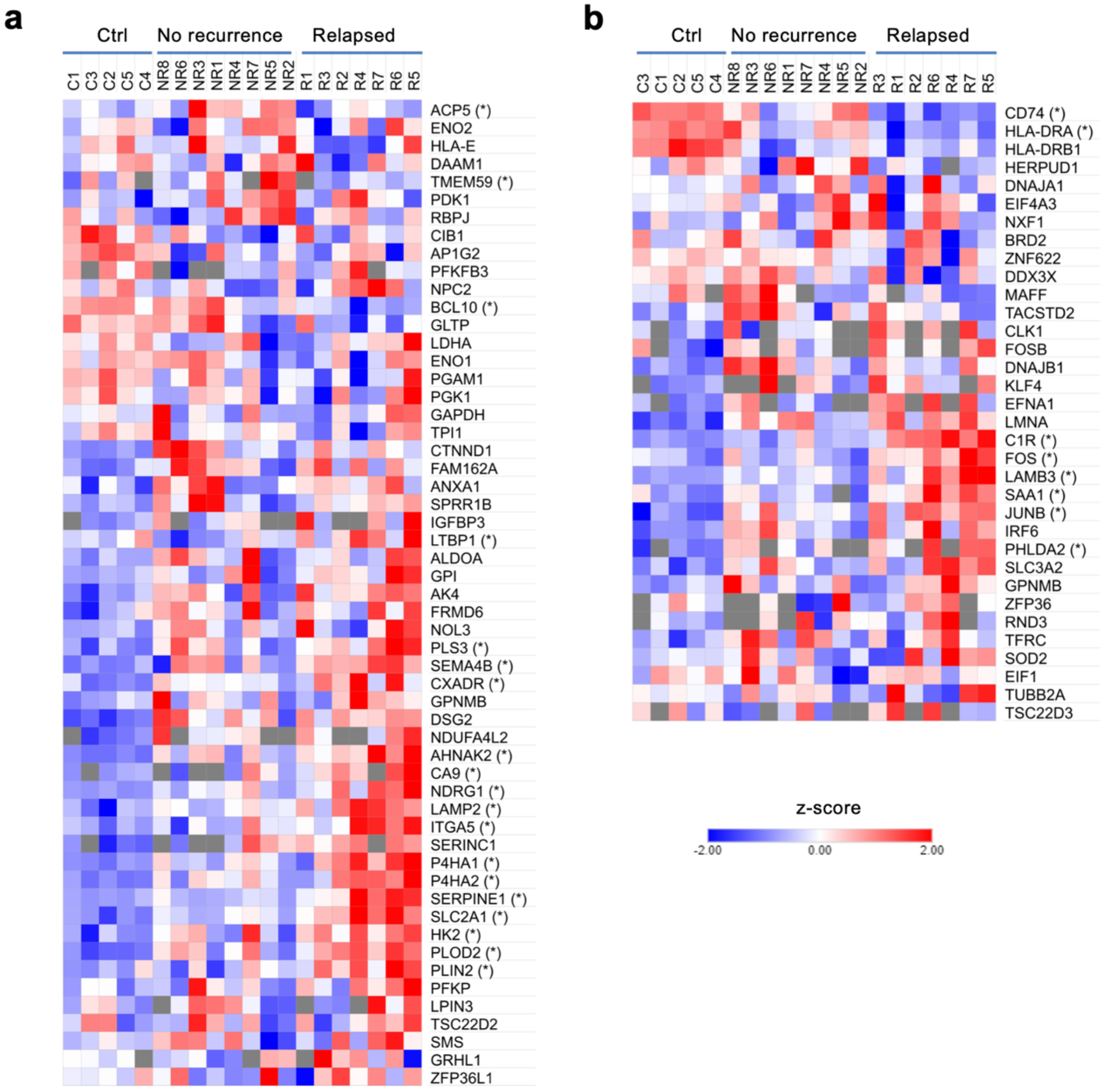

Supplementary Figure 1. Heatmap of functional gene signatures for hypoxia (a) and stress response (b). Significantly increased or decreased proteins were marked with an asterisk (*) (p-value < 0.1 between the non-recurrence (NR) and recurrence (R) groups). n = 8 for the NR group, and n = 7 for the R group.

a

b

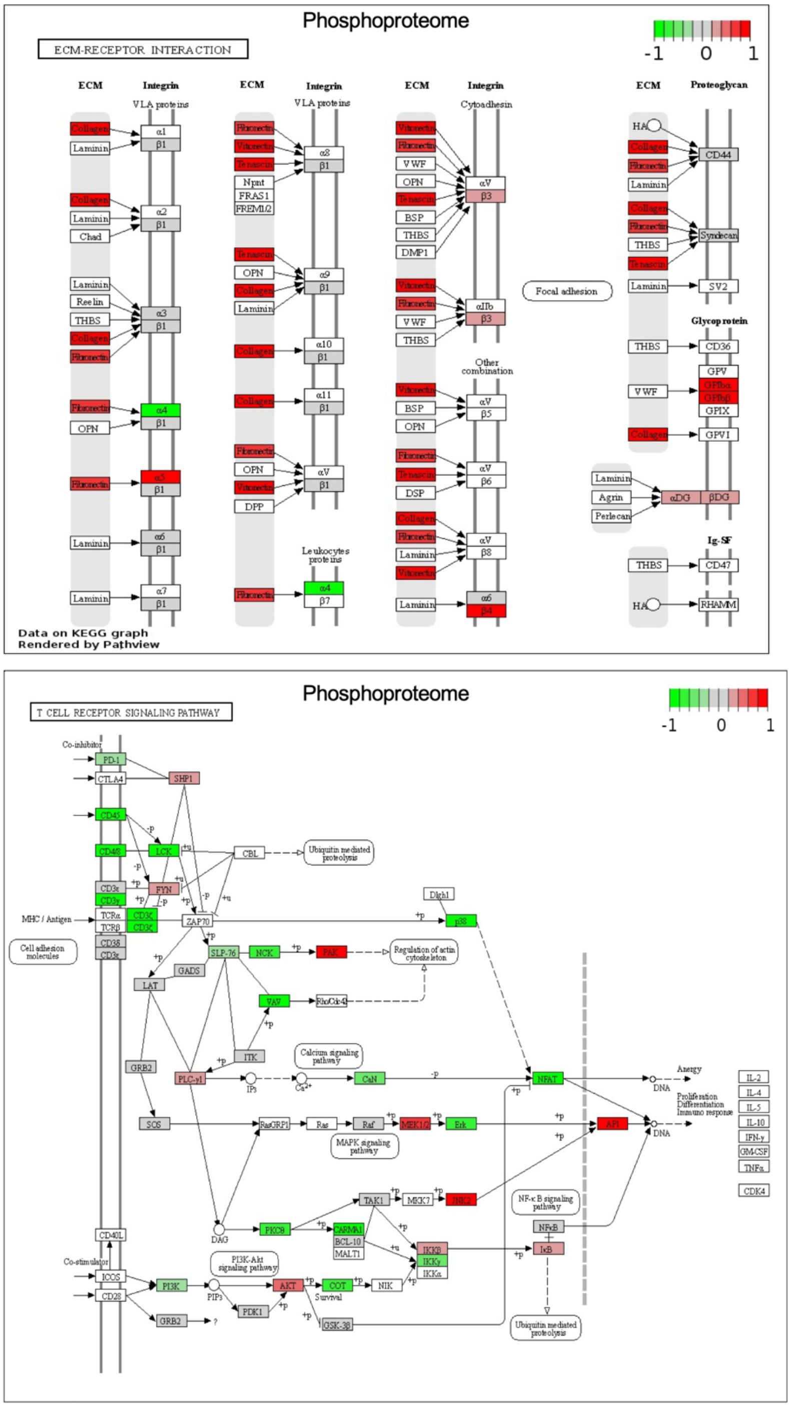

Supplementary Figure 2. Signaling pathways altered in the recurrent HNSCC. Shown are extracellular matrix (ECM)-receptor interaction (**a**) and T cell receptor (TCR) signaling (**b**) signaling pathways annotated with increased (red) and decreased (green) phosphorylation in the recurrent tumors. See also Supplementary Tables 7 and Supplementary Data 1.

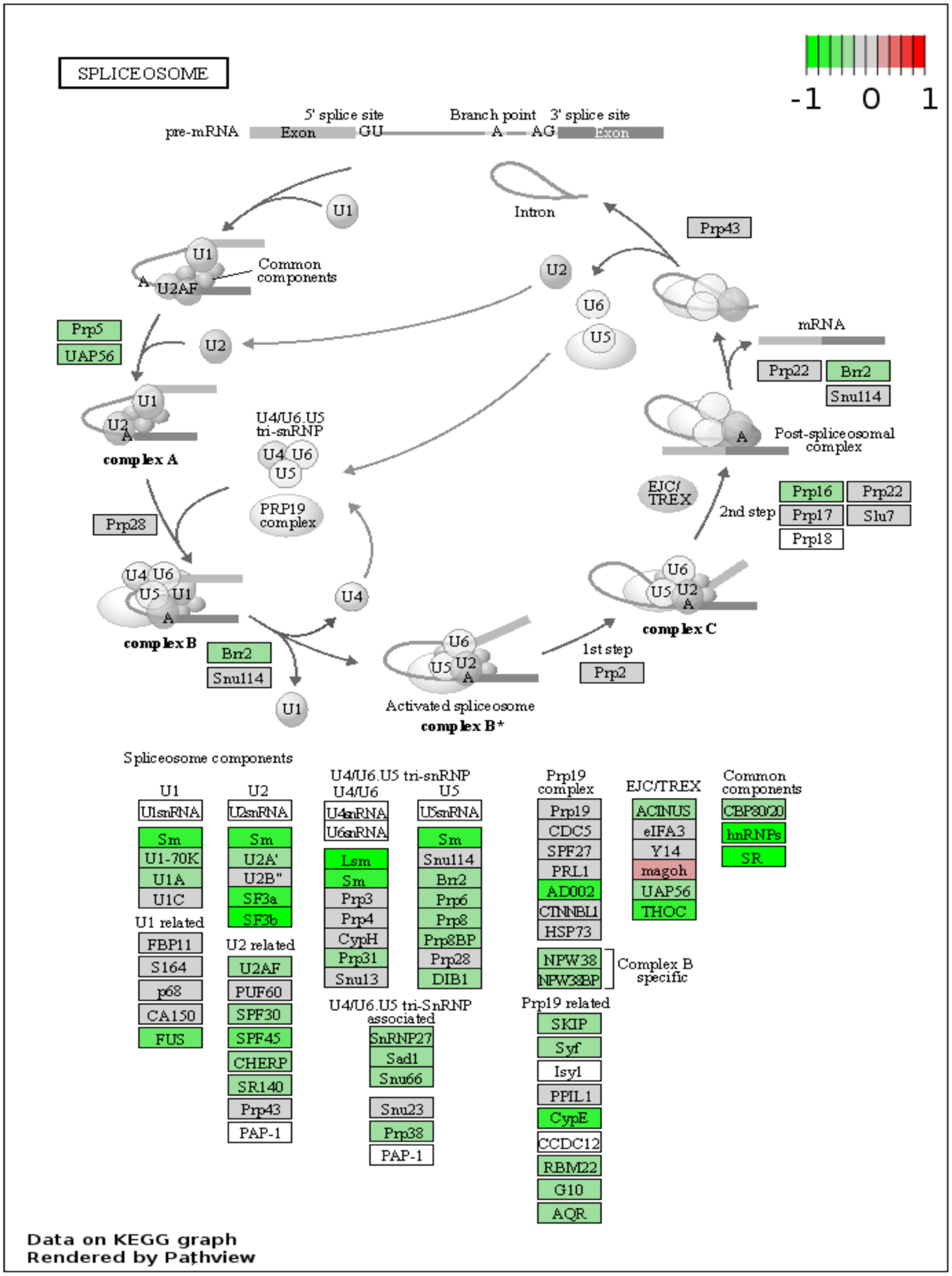

Supplementary Figure 3. The RNA splicesome is down-regulated in the recurrent tumor. The spliceosome components with significantly increased or decreased protein levels in the recurrent tumors are denoted in red or green based on the proteome data.

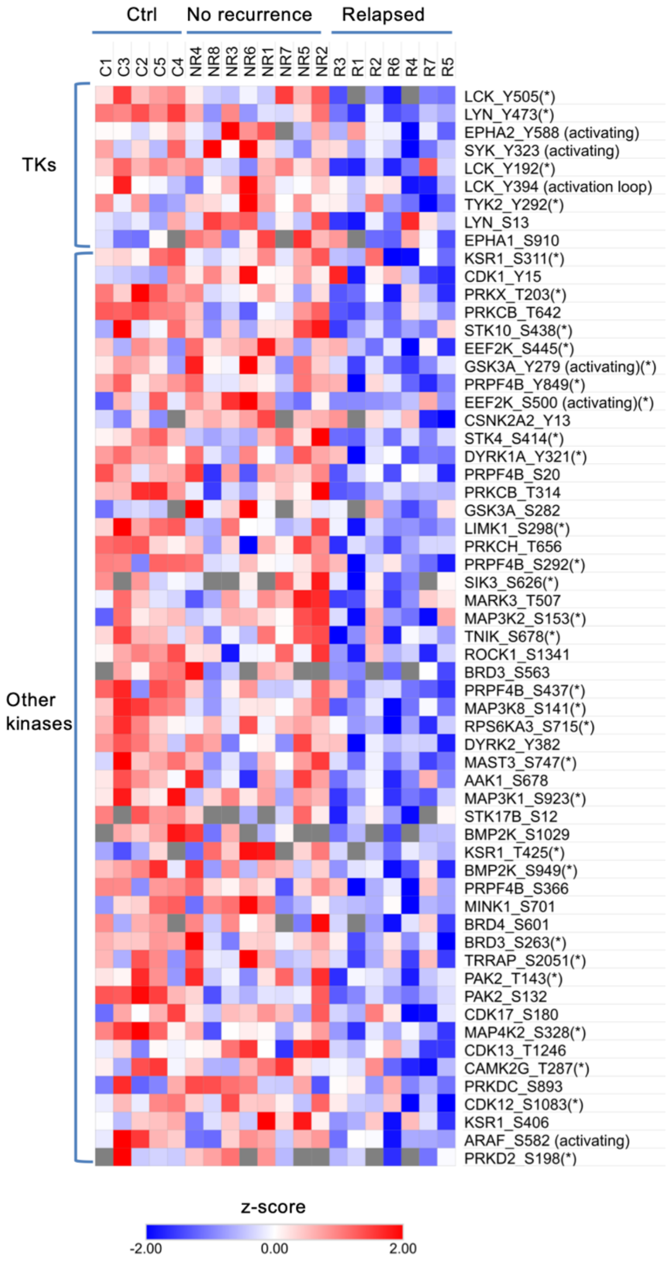

Supplementary Figure 4. Heatmap of protein kinases showing decreased phosphorylation in the recurrent HNSCC samples. Kinase phosphorylation sites with a significant difference ($p < 0.1$ between NR and R groups) are listed. The phosphosites with an asterisk (*) indicates $p < 0.05$ between the two groups. n = 8 for the NR group, and n = 7 for the R group.
