# Supplementary material for: Proteome and phosphoproteome signatures of recurrence for HPV+ head and neck squamous cell carcinoma
Source: Commun Med (Lond). 2022 Jul 30;2:95. doi: 10.1038/s43856-022-00159-8 (PMC9338924; doi:10.1038/s43856-022-00159-8)
Supplement: Supplementary file 5 — Description of Additional Supplementary Files [file 43856_2022_159_MOESM5_ESM.pdf]

Description of Additional Supplementary Files

File Name: Supplementary Data 1

Description: A list of phosphosites used to draw the T cell receptor signaling pathway.

File Name: Supplementary Data 2

Description: Protein and phosphosite intensities from the mass spectrometry analysis

File Name: Supplementary Data 3

Description: Source data for the main figures in this manuscript.
