# Supplementary material for: Proteome and phosphoproteome signatures of recurrence for HPV+ head and neck squamous cell carcinoma
Source: Commun Med (Lond). 2022 Jul 30;2:95. doi: 10.1038/s43856-022-00159-8 (PMC9338924; doi:10.1038/s43856-022-00159-8)
Supplement: Supplementary file 7 — Reporting Summary [file 43856_2022_159_MOESM7_ESM.pdf]

nature research

Corresponding author(s): Shawn SC Li, Anthony C Nichols

Last updated by author(s): Jun 7, 2022

Reporting Summary

Nature Research wishes to improve the reproducibility of the work that we publish. This form provides structure for consistency and transparency in reporting. For further information on Nature Research policies, see our Editorial Policies and the Editorial Policy Checklist.

Statistics

For all statistical analyses, confirm that the following items are present in the figure legend, table legend, main text, or Methods section.

n/a	Confirmed	
☐	☒	The exact sample size (*n*) for each experimental group/condition, given as a discrete number and unit of measurement
☐	☒	A statement on whether measurements were taken from distinct samples or whether the same sample was measured repeatedly
☐	☒	The statistical test(s) used AND whether they are one- or two-sided<br>*Only common tests should be described solely by name; describe more complex techniques in the Methods section.*
☒	☐	A description of all covariates tested
☒	☐	A description of any assumptions or corrections, such as tests of normality and adjustment for multiple comparisons
☐	☒	A full description of the statistical parameters including central tendency (e.g. means) or other basic estimates (e.g. regression coefficient) AND variation (e.g. standard deviation) or associated estimates of uncertainty (e.g. confidence intervals)
☐	☒	For null hypothesis testing, the test statistic (e.g. *F*, *t*, *r*) with confidence intervals, effect sizes, degrees of freedom and *P* value noted<br>*Give P values as exact values whenever suitable.*
☒	☐	For Bayesian analysis, information on the choice of priors and Markov chain Monte Carlo settings
☒	☐	For hierarchical and complex designs, identification of the appropriate level for tests and full reporting of outcomes
☐	☒	Estimates of effect sizes (e.g. Cohen's *d*, Pearson's *r*), indicating how they were calculated

Our web collection on statistics for biologists contains articles on many of the points above.

Software and code

Policy information about availability of computer code

Data collection	Thermo Scientific Xcalibur ver. 4.3.73.11
Data analysis	FragPipe ver. 16.0, Perseus ver. 1.6.14.0, VolcaNoseR server, Metascape server, Pathview Web, Morpheus server, Cytoscape 3.9.0, GraphPad Prism 9

For manuscripts utilizing custom algorithms or software that are central to the research but not yet described in published literature, software must be made available to editors and reviewers. We strongly encourage code deposition in a community repository (e.g. GitHub). See the Nature Research guidelines for submitting code & software for further information.

Data

Policy information about availability of data

All manuscripts must include a data availability statement. This statement should provide the following information, where applicable:

- Accession codes, unique identifiers, or web links for publicly available datasets
- A list of figures that have associated raw data
- A description of any restrictions on data availability

The mass spectrometry proteomics/phosphoproteomics data have been deposited to the ProteomeXchange Consortium via the PRIDE partner repository with the dataset identifier PXD030343.

Field-specific reporting

Please select the one below that is the best fit for your research. If you are not sure, read the appropriate sections before making your selection.

☒ Life sciences ☐ Behavioural & social sciences ☐ Ecological, evolutionary & environmental sciences

For a reference copy of the document with all sections, see nature.com/documents/nr-reporting-summary-flat.pdf

Life sciences study design

All studies must disclose on these points even when the disclosure is negative.

Sample size	We collected samples from HNSCC patients since 2010 at London Health Science Centre, Victoria Hospital, Canada, and we were able to obtain 15 sample materials (8 without recurrence, 7 with recurrence) suitable for proteomic analysis. 5 healthy control samples were also included
Data exclusions	No data were excluded
Replication	The mass spectrometry samples contain 6 technical replicate samples to confirm measurement and sample handling reproducibility
Randomization	The 20 samples were randomized into three 11-plex TMT channels to minimize systematic errors caused by the batch effect
Blinding	This is an observational study and blinding was not performed

Reporting for specific materials, systems and methods

We require information from authors about some types of materials, experimental systems and methods used in many studies. Here, indicate whether each material, system or method listed is relevant to your study. If you are not sure if a list item applies to your research, read the appropriate section before selecting a response.

Materials & experimental systems

n/a	Involved in the study	
☒	☐	Antibodies
☐	☒	Eukaryotic cell lines
☒	☐	Palaeontology and archaeology
☒	☐	Animals and other organisms
☐	☒	Human research participants
☒	☐	Clinical data
☒	☐	Dual use research of concern

Methods

n/a	Involved in the study	
☒	☐	ChIP-seq
☒	☐	Flow cytometry
☒	☐	MRI-based neuroimaging

Eukaryotic cell lines

Policy information about cell lines

Cell line source(s)	93-VU-147T was a gift from VU Medical Center, Amsterdam
Authentication	The cell line was not authenticated. The cell line was used solely to globally increase the MS1 intensities of tyrosine-phosphorylated peptides in phosphoproteomic data collection
Mycoplasma contamination	The cell line was tested negative for mycoplasma contamination
Commonly misidentified lines (See ICLAC register)	none

Human research participants

Policy information about studies involving human research participants

Population characteristics	Of the 15 patients, 14 are male, 9 has smoking history, the median age is 60 (between 45 and 80 years old)
Recruitment	Since this was an observational study, recruitment was driven by availability of the material.
Ethics oversight	The study was approved by the Research Ethics Boards at Western University (REB 7182) and informed written consent was obtained from each patient

Note that full information on the approval of the study protocol must also be provided in the manuscript.
